# Formononetin attenuates atherosclerosis via regulating interaction between KLF4 and SRA in apoE^-/-^ mice

**DOI:** 10.7150/thno.38115

**Published:** 2020-01-01

**Authors:** Chuanrui Ma, Ronglin Xia, Shu Yang, Lipei Liu, Jing Zhang, Ke Feng, Yuna Shang, Jingtian Qu, Lingwei Li, Ning Chen, Shixin Xu, Wenwen Zhang, Jingyuan Mao, Jihong Han, Yuanli Chen, Xiaoxiao Yang, Yajun Duan, Guanwei Fan

**Affiliations:** 1 First Teaching Hospital of Tianjin University of Traditional Chinese Medicine, Tianjin, China;; 2 Tianjin Key Laboratory of Translational Research of TCM Prescription and Syndrome, Tianjin, China;; 3 State Key Laboratory of Modern Chinese Medicine, Tianjin University of Traditional Chinese Medicine, Tianjin, China;; 4 Department of Endocrinology, The Second Clinical Medical College of Jinan University, Shenzhen People's Hospital, Shenzhen, China;; 5 College of Life Sciences, Nankai University, Tianjin; China;; 6 School of Food and Biological Engineering, Hefei University of Technology, Hefei, China;; 7 Tianjin Hospital, Tianjin, China;; 8 School of Materials Science and Engineering, Tianjin University, Tianjin, China;; 9 Tianjin Central Hospital of Obstetrics and Gynecology, Tianjin, China

**Keywords:** formononetin, cholesterol uptake, atherosclerosis, inflammation, foam cells, Krüppel-like factor 4, Scavenger receptor A1

## Abstract

**Background and Purpose**: Atherosclerosis is an underlying cause of coronary heart disease. Foam cell, a hallmark of atherosclerosis, is prominently derived from monocyte-differentiated macrophage, and vascular smooth muscle cells (VSMCs) through unlimitedly phagocytizing oxidized low-density lipoprotein (oxLDL). Therefore, the inhibition of monocyte adhesion to endothelium and uptake of oxLDL might be a breakthrough point for retarding atherosclerosis. Formononetin, an isoflavone extracted from *Astragalus membranaceus*, has exhibited multiple inhibitory effects on proatherogenic factors, such as obesity, dyslipidemia, and inflammation in different animal models. However, its effect on atherosclerosis remains unknown. In this study, we determined if formononetin can inhibit atherosclerosis and elucidated the underlying molecular mechanisms.

**Methods**: ApoE deficient mice were treated with formononetin contained in high-fat diet for 16 weeks. After treatment, mouse aorta, macrophage and serum samples were collected to determine lesions, immune cell profile, lipid profile and expression of related molecules. Concurrently, we investigated the effect of formononetin on monocyte adhesion, foam cell formation, endothelial activation, and macrophage polarization *in vitro* and *in vivo*.

**Results**: Formononetin reduced *en face* and aortic root sinus lesions size. Formononetin enhanced lesion stability by changing the composition of plaque. VSMC- and macrophage-derived foam cell formation and its accumulation in arterial wall were attenuated by formononetin, which might be attributed to decreased SRA expression and reduced monocyte adhesion. Formononetin inhibited atherogenic monocyte adhesion and inflammation. KLF4 negatively regulated the expression of SRA at transcriptional and translational level.

**Conclusions**: Our study demonstrate that formononetin can substantially attenuate the development of atherosclerosis via regulation of interplay between KLF4 and SRA, which suggests the formononetin might be a novel therapeutic approach for inhibition of atherosclerosis.

## Introduction

Atherosclerosis has been characterized as chronic inflammation and cholesterol deposition within arteries, which is one of the underlying causes of coronary heart disease 1. Lipid deposition can induce inflammatory response, which in turn promotes the monocyte adhesion and infiltration, accelerates cholesterol accumulation, and further amplifies inflammation in circulation 2-4. Lipid-overladen foam cells are the prominent part of atherosclerotic lesions 5 and mainly derived from VSMCs and monocyte-differentiated macrophages 6,7. Formation of foam cells depends on the homeostasis between lipid uptake and efflux, which are predominantly determined by macrophage scavenger receptors (MSRs) such as SRA, CD36, SRBI, LOX-1, responsible for lipid uptake 8-11; and cholesterol transporters such as ABCA1 and ABCG1, responsible for cholesterol efflux 12. Excessive cholesterol uptake and decreased efflux in macrophage and VSMCs can result in foam cell formation 13, suggesting that the homeostasis between cholesterol uptake and efflux is a key factor for resistance of atherosclerosis development. Unfortunately, as a major source of foam cells, VSMCs express lower ABCA1 6 and higher SRA 14, thereby synergistically exacerbating the foam cell differentiation. These studies indicate that effective intervention on MSR expression may protect macrophages and VSMCs against foam cell formation. Beside the MSR-mediated phagocytosis function, MSR also participates in regulation of macrophage phenotype shift 15 and inflammation 16,17, indicating that SRA may regulate the inflammatory response at the initiation and progression of atherosclerosis.

Synthetic liver X receptor (LXR) ligands can potently inhibit atherosclerosis, but dramatically induce hepatic steatosis and hypertriglyceridemia 18-22, which hinders its application for atherosclerosis treatment. We have ever attempted to remove the adverse effect of LXR ligand by strategy of coadministration with other drugs such as MEK1/2 inhibitor or AMPK activator 23,24. Despite the efforts was paid on eliminating the undesirable side effect of LXR ligand, but given the potential safety risks and clinical limitation, the more novel strategies or drug candidates for atherosclerosis treatment are still necessary. As is reported, a specific SRA blocker has shown potential for targeting the atherosclerotic plaque 25. Subsequently, we concentrate on the inhibition of MSR-mediated lipoprotein uptake during foam cell formation. However, the underlying mechanism of regulating MSR expression remains unknown. Krüppel-like factor 4 (KLF4), a transcription factor, is a member of the KLF family 26. Noticeably, KLF4 plays an important role in atherosclerosis. SMC-specific conditional knockout of KLF4 reduces lesion size 27. However, other studies indicate that KLF4 exerts antiatherogenic effect 28-30. Moreover, KLF4-deficient macrophages demonstrated increased proinflammatory gene expression, indicating that KLF4 is essential for promoting M2 polarization 31.The above studies show that both MSR and KLF4 play important roles in atherogenesis; however, whether the interplay occurs between MSR and KLF4 remains unknown.

Formononetin is a major flavonoid component extracted from *Astragalus membranaceus* that have protective effects on cardiovascular disease 32,33. It is reported that formononetin can protect against obesity, a contributor to atherogenesis 34, through activating AMPK and PPARγ pathway 35,36. Noticeably, activation of AMPK can modulate the expression of KLF4 37 and inhibit atherosclerosis 24. These results indicate that formononetin may regulate KLF4 expression during atherosclerosis development. Given the role of KLF4 and SRA in atherogenesis, we postulate that formononetin may be antiatherogenic, in part, through regulation of KLF4-SRA signaling. In this study, we investigated the effect of formononetin on atherosclerosis *in vivo* and *in vitro*. In proatherogenic apoE^-/-^ mice, formononetin significantly attenuates atherosclerosis. Moreover, KLF4 acts as a suppressor on expression of SRA. Formononetin reduces the binding between endothelial cells and monocytes; inhibits macrophage- and VSMC-derived foam cell formation; and inhibits the expression of SRA by increasing KLF4 expression as well as nuclear translocation. Therefore, the antiatherogenic properties of formononetin are partially through regulation of KLF4-SRA axis.

## Results

### Formononetin inhibits atherosclerosis and enhances lesion plaque stability

**Figure 1:**
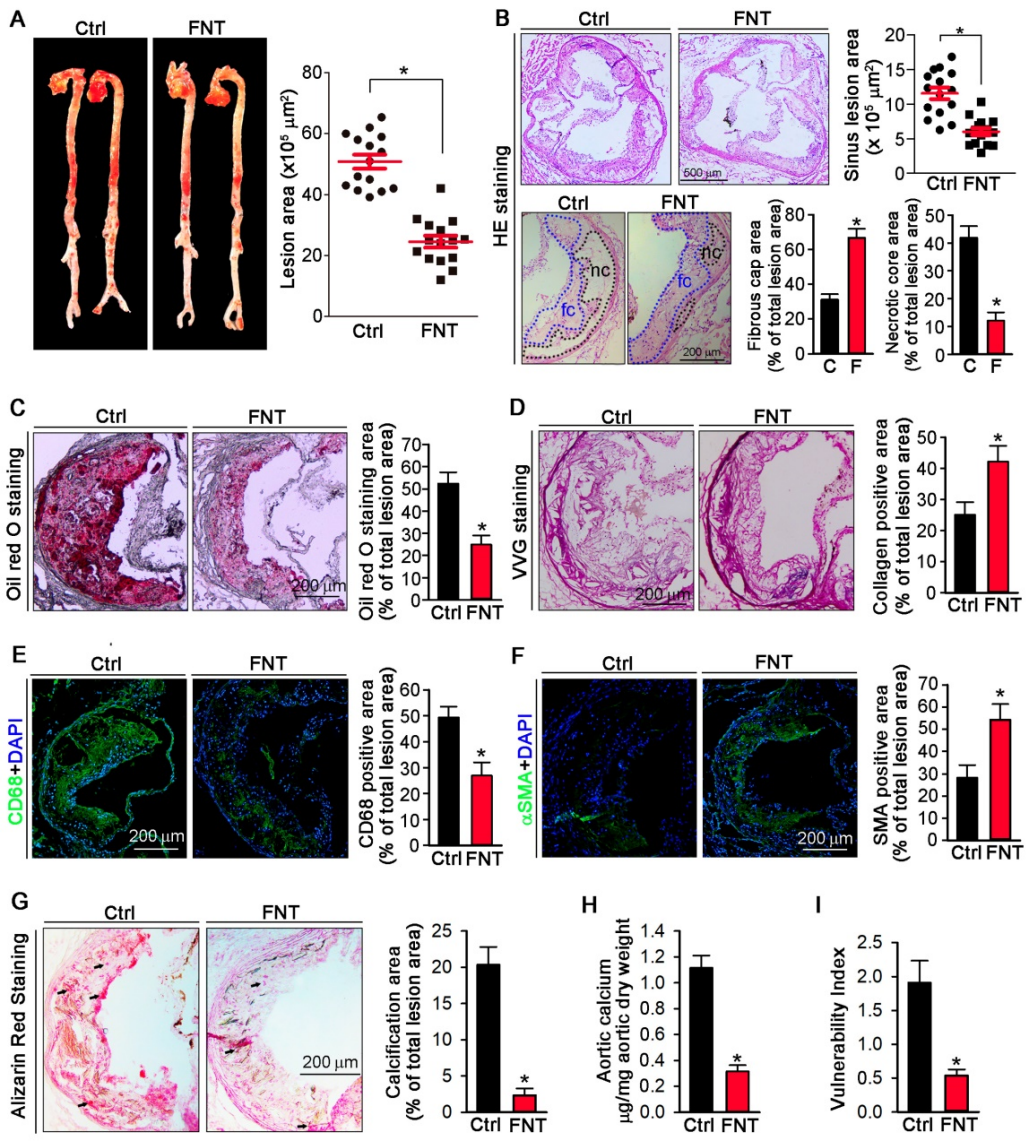
** Formononetin inhibits atherosclerosis and enhances plaque stability.** ApoE^-/-^ mice in 2 groups (15/group) received the following treatment for 16 weeks: Control, HFD; FNT, HFD containing formononetin (FNT) (10 mpk). (**A**) After treatment, aortas were isolated for determination of lesions in *en face* aortas by Oil Red O staining and quantified by a computer assisted image analysis protocol. Lesion areas were expressed as μm^2^, n=15. The following assays were performed in aortic root cross sections: (**B**) Haematoxylin and eosin staining followed by quantitative analysis of sinus lesions (upper), necrotic core area and fibrous cap area (bottom) in aortic root cross sections. nc: necrotic cores marked by black dotted line; fc: fibrous cap marked by blue dotted line; Lesion areas were expressed as μm^2^, n=15. (**C-F**) Representative photomicrographs (left) and quantification (right) of aortic root sections stained with Oil Red O (C), Verhoeff-Van Gieson (D), CD68 (E) and αSMA (F) in atherosclerotic plaque, n=15. (**G**) Alizarin Red S staining for calcification (indicated by black arrows) and quantification of calcification positive areas, n=15. (**H)** Evaluation of total calcium extracted from whole aorta by calcium assay kit, n=5. (**I**) Vulnerability index of plaques, n=15. Data are presented as mean ± SEM, *P<0.05, significantly different from control. Ctrl: control; FNT: formononetin.

To determine if formononetin can inhibit atherosclerosis, apoE^-/-^ mice were fed HFD or HFD containing formononetin for 16 weeks. Figures 1A showed that *en face* aortic lesions were inhibited by 43% after formononetin treatment. Correspondingly, Figures 1B (upper) demonstrated that formononetin resulted in 48% reduction in areas of atherosclerotic plaques in the aortic sinus. The decrease of necrotic core size can destabilize the plaques, whereas the increase of fibrous cap area can reduce the vulnerability of plaque. In this study, necrotic core area was markedly reduced while fibrous cap area was increased by formononetin (Figure 1B, bottom), which may be attributed to the anti-apoptotic effect of formononetin (Figure S2). We further determined the effects of formononetin on plaque composition. Lipid deposition in plaques was less in formononetin-treated mice (Figure 1C). In addition, VVG staining demonstrated that formononetin increased collagen content (Figure 1D), which contributed the plaque stabilization. Furthermore, formononetin significantly reduced the macrophage accumulation, as indicated by immunostaining with CD68 (Figure 1E). In contrast, content of VSMCs, the main cell type in lesion caps to stabilize lesion plaques, was substantially increased by formononetin, as indicated by immunostaining with αSMA (Figure 1F). Consequently, the vulnerability index of the plaque was reduced by formononetin (Figure 1I). Moreover, we evaluated the calcium deposition in plaque, an important contributor to vulnerability of plaque; and observed that formononetin substantially inhibited calcification in aortic root (Figure 1G), which was further confirmed by the quantitation of calcium content in whole aortas (Figure 1H). Taken together, the data suggested that formononetin can retard the atherosclerotic lesion development and enhance plaque stability.

### Formononetin inhibits macrophage/VSMC-derived foam cell formation by reduction of cholesterol uptake

**Figure 2:**
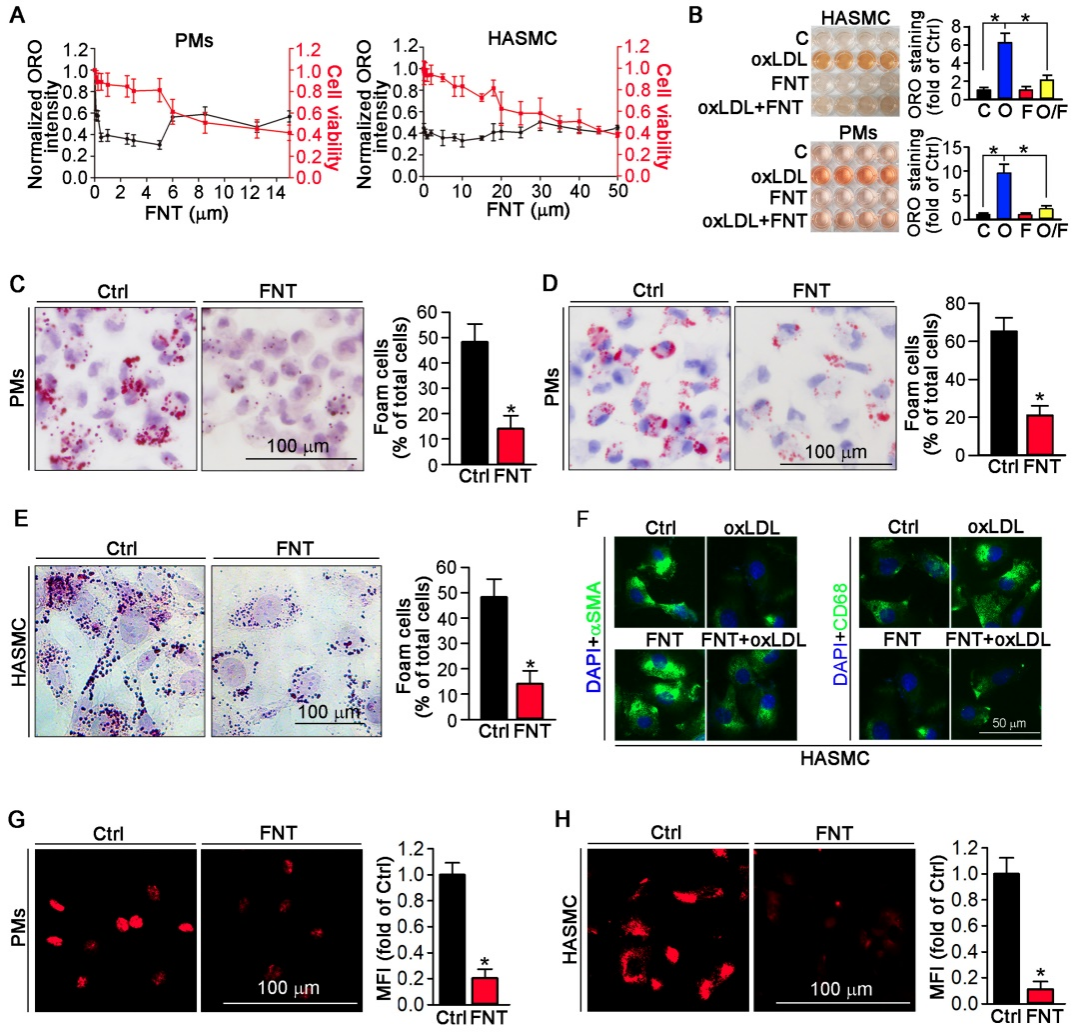
** Formononetin inhibits lipid accumulation in HASMCs and PMs.** (**A**) Evaluation of the dose-dependent lipid-reducing effects and cytotoxicity of formononetin in peritoneal macrophages (PMs) and HASMCs using Oil Red O (ORO) staining and MTT assay, n=5. (**B**) Images (Left) and quantitation (Right) of the extracted ORO dyes from the stained PMs and HASMCs that were treated with oxLDL (O), formononetin (FNT, F) and oxLDL together with FNT (O/F), n=5. (**C**) PMs isolated from mice used in Figure 1 were stained with ORO to assess formation of foam cells (>10 lipid droplets/cell, >10 fields/sample), n=5. (**D, E**) PMs isolated from untreated apoE^-/-^ mice (D) and HASMCs (E) were treated with oxLDL or/and FNT, followed by ORO staining to assess formation of foam cells, n=5. (**F**) Evaluation of CD68 and αSMA expression after incubation with oxLDL or/and formononetin by immunofluorescence staining, n=5. (**G, H**) Cholesterol uptake of PMs and HASMCs were determined by Dil-oxLDL after treatment as indicated, n=5. Data are presented as mean ± SEM, *P<0.05, significantly different as indicated.

**Figure 3:**
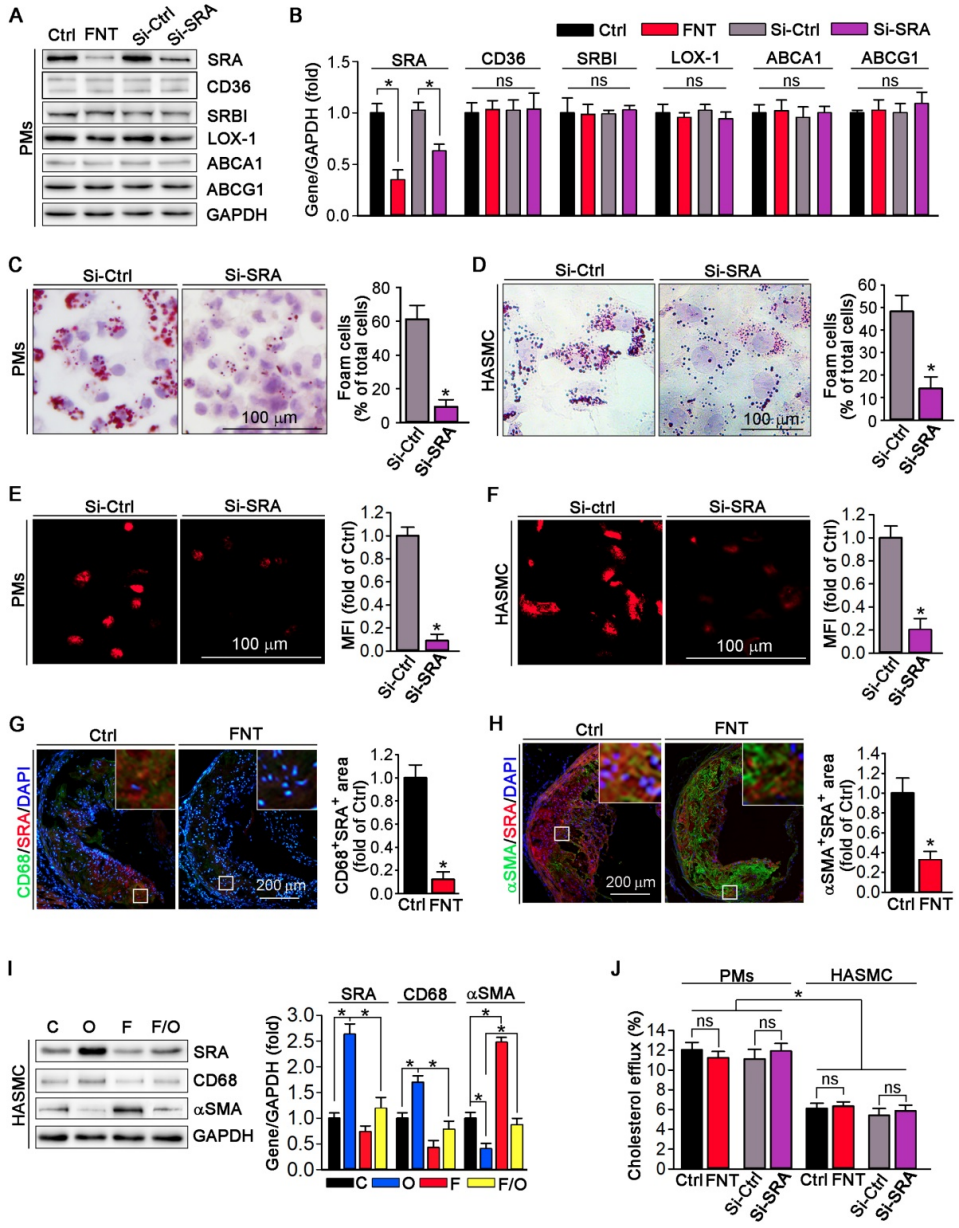
** Function of SRA in formononetin-inhibited lipid overload in HASMCs and macrophages.** (**A, B**) Expression of SRA, CD36, SRBI, LOX1, ABCA1, and ABCG1 in protein of PMs after formononetin or siSRA treatment by Western blot, followed by quantification, n=5. (**C, D**) After knockdown of SRA by siRNA, PMs and HASMCs were treated with oxLDL (100 μg mL^-1^) and followed by ORO staining (left) and corresponding quantitative analysis (right), n=5. (**E, F**) Cholesterol uptake of PMs and HASMCs were determined by Dil-oxLDL after treatment as indicated, n=5. (**G, H**) Aortic root cross sections from mice used in Figure 1 were conducted co-immunofluorescent staining with anti-SRA and CD68 or αSMA antibodies with quantitative analysis of CD68^+^SRA^+^ or αSMA^+^ SRA^+^ area, n=5. (**I**) CD68, SRA and αSMA in HASMCs were evaluated by Western blot after treatment as indicated, n=5. (**J**) Cholesterol efflux of PMs and HASMCs were assessed after treatment as indicated, n=5. Data are presented as mean ± SEM, *P<0.05, significantly different as indicated; ns: not significantly different. Ctrl or C: control; FNT or F: formononetin; O: oxLDL.

To determine whether formononetin can inhibit the lipid burden in macrophage and VSMCs. Peritoneal macrophages (PMs) and human aortic smooth muscle cells (HASMCs) were treated with a series of concentrations of formononetin for 24 h in presence of oxLDL. Formononetin significantly lowered the cellular lipid droplets at as low as 1-5 μM and maintained potent lipid-lowering effect at up to 5 μM while ∼80% of the cells were of viability (Figure 2A). Similar results were obtained when ORO-stained cells were directly photographed (Figure 2B). *In vivo*, Figure 2C demonstrated that HFD lead ~48% of PMs to differentiate into foam cells. Intriguingly, formononetin decreased the rate to ~14%, suggesting the potent inhibition of foam cell formation. *In vitro*, formononetin significantly decreased the oxLDL-induced foam cells (Figure 2D). We determined whether formononetin can retard oxLDL-induced lipid accumulation in HASMC. As shown in figure 2E, lipid overload was significantly reduced by formononetin. We further determined the effect of formononetin on HASMC phenotype switching. Formononetin significantly antagonized the oxLDL-induced CD68 expression and partially reversed the oxLDL-reduced αSMA expression (Figure 2F). Furthermore, cholesterol uptake and efflux assays revealed that formononetin significantly reduced the level of cholesterol uptake by PMs and HASMCs (Figure 2G-H); whereas had no effect on cholesterol efflux (Figure 3J), indicating that formononetin can reduce lipid deposition particularly by inhibiting cholesterol uptake. Taken together, inhibition of macrophage- and VSMC-derived foam cell formation by formononetin was through reducing cholesterol uptake and preventing VSMC to change to macrophage-like cells, which confirmed the *in vivo* inhibitory effect of formononetin on foam cell formation.

### Formononetin inhibits lipid accumulation in macrophage and VSMC by inhibition of SRA-mediated cholesterol uptake

We evaluated the expression of scavenger receptors and cholesterol transporters in PMs by Western blot; and observed that formononetin significantly inhibited the expression of SRA, whereas had no effect on CD36, SRBI, LOX-1, ABCA1 and ABCG1 expression (Figure 3A), indicating that formononetin may reduce the cholesterol uptake particularly by inhibition of SRA. Furthermore, cellular lipid burden (Figure 3C-D) and cholesterol uptake (Figure 3E-F) in PMs and HASMC were markedly reduced after knockdown of SRA. In macrophages, modified LDLs bound to SRA are internalized through clathrin-dependent endocytosis, which is decided by activity of GTPase dynamin. Endocytosis of oxLDL was reduced by dynasore (the inhibitor of GTPase dynamin), which cannot be further reduced by formononetin (Figure S3), indicating that inhibition of SRA-mediated ox-LDL endocytosis by formononetin may be dynamin-mediated. To further determine the effect of formononetin on SRA expression *in situ*, we assessed the expression of SRA in macrophages and VSMCs within plaques by dual immunofluorescent staining with anti-SRA and CD68/αSMA antibodies. The results exhibited significant reduction in CD68^+^ SRA^+^ and αSMA^+^ SRA^+^ area (Figure 3G-H), indicating that formononetin can reduce the SRA expression in macrophages and VSMCs within plaques. In addition, we further detected the effect of formononetin on VSMCs phenotype by Western blot; and observed that oxLDL-induced expression of macrophage marker CD68 was markedly inhibited, while the VSMC marker αSMA was partially restored by formononetin (Figure 3I), which was corresponding with immunofluorescent staining (Figure 2F). Simultaneously, formononetin significantly antagonized the oxLDL-induced SRA expression in HASMC at protein level (Figure 3I), suggesting that reduction of SRA by formononetin may be associated with inhibition of HASMC toward macrophage-like cell and foam cell. Collectively, these results demonstrated that formononetin can suppress oxLDL-driven foam cell formation, mechanistically, by inhibition of SRA-mediated cholesterol uptake.

### Formononetin blocks atherogenic monocytes adhesion to HUVECs and aortic endothelium partially through reducing SRA expression

**Figure 4:**
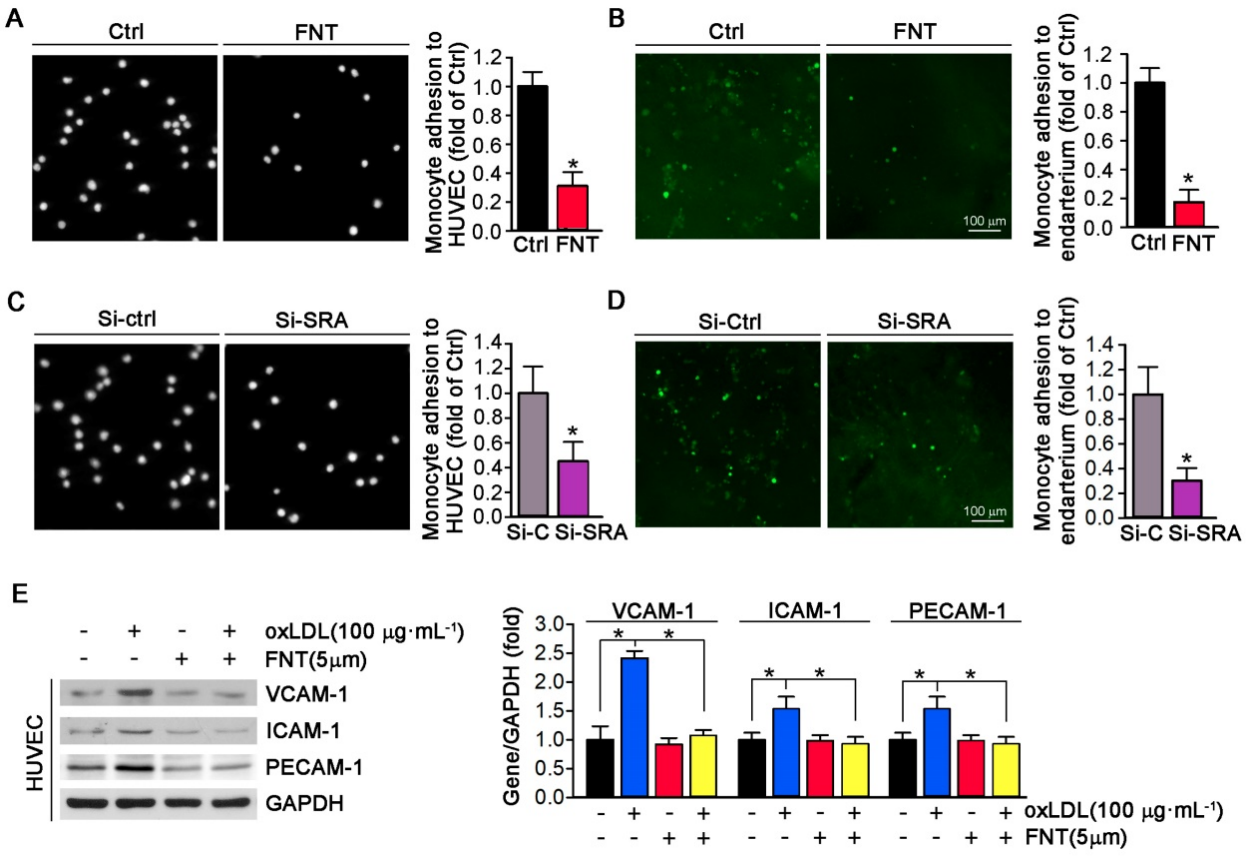
** Formononetin inhibits monocyte adhesion to ECs and endothelium.** (**A**) HUVECs in 24-well plates and THP-1 cells were pretreated with oxLDL (100 μg mL^-1^) for 2 h followed by addition of formononetin overnight. After incubation with oxLDL, THP-1 cells were labeled with CFSE, and then added to HUVECs and co-incubated for 1 h. The image of adherent THP-1 cells were captured with a microscope. The number of adherent THP-1 cells in control group (treated with oxLDL alone) was defined as 1. The fold changes were obtained by calculating the ratio of adherent cells in the corresponding group to that in the control group, n=5. (**B**) Aorta isolated from untreated apoE^-/-^ mice and THP-1 cells were pretreated with oxLDL (100 μg mL^-1^) for 2 h followed by addition of formononetin overnight. CFSE-labeled THP-1 cells were then added to HUVECs and co-incubated for 1 h. The evaluation of adherent THP-1 cells was performed as described in (A), n=5. (**C, D**) After knockdown of SRA, THP-1 cells were labeled with CFSE and then incubated with oxLDL-pretreated HUVEC or aortic endothelium. The evaluation of adherent THP-1 cells was conducted as described in (A), n=5. (**E**) Expression of VCAM-1, ICAM-1 and PECAM-1 protein in HUVECs was determined by western blot (left) followed by quantitative analysis (right), n=5. Data are presented as mean ± SEM, *P<0.05, significantly different as indicated.

The accumulation of macrophage-derived foam cells within artery wall is mainly determined by adhesion of monocytes to endothelium and infiltration. To determine whether formononetin can directly inhibit monocyte adhesion to endothelial cells, we performed *in vitro* and *in ex vivo* experiment. As shown in Figure 4A, formononetin substantially reduced the number of THP-1 cells adhering to HUVECs (reduction to ∼30% of control). In addition, formononetin also inhibited the monocytes adhesion to endothelium of aorta that isolated from apoE^-/-^ mice *in ex vivo* experiment (reduction to ∼16% of control) (Figure 4B), which further supported the inhibitory effect of formononetin on monocytes adhesion. Subsequently, we explored the molecular mechanisms associated with the formononetin-inhibited monocyte adhesion. We tested the function of SRA in monocytes adhesion. Interestingly, SRA knockdown in THP-1 cells significantly reduced the number of THP-1 cells adhering to HUVECs (reduction to ∼42% of control) (Figure 4C) and aortic endothelium (reduction to ∼30% of control) (Figure 4D). In comparison, the number of formononetin-treated THP-1 cells adhering to HUVECs and aortic endothelium was less than that of siSRA-treated THP-1 cells (reduction to ∼30 % vs ∼42 %, adhesion to HUVECs; reduction to ∼16% vs ∼30%, adhesion to aortic endothelium), which suggested the inhibitory effect of formononetin was more remarkable than that of siSRA, indicating that other associated molecules may be involved in this progress. Hence, we further determined expression of VCAM-1, ICAM-1 and PECAM-1 in HUVECs, three classical molecules for monocyte adhesion; and observed that expressions of these molecules were significantly reduced by formononetin in oxLDL-treated HUVECs (Figure 4E), which indicated that reduction of these molecules may be partially responsible for formononetin-inhibited monocyte adhesion. Moreover, formononetin did not affect the migration of HUVEC and expression of MMP2/MM9 (Figure S1). Altogether, formononetin can inhibit the monocyte adhesion by reducing the expression of SRA in monocyte and VCAM-1, ICAM-1 and PECAM-1 in HUVECs.

### Formononetin inhibits the inflammation and promotes macrophage phenotypic transition to M2 anti-inflammatory type

**Figure 5:**
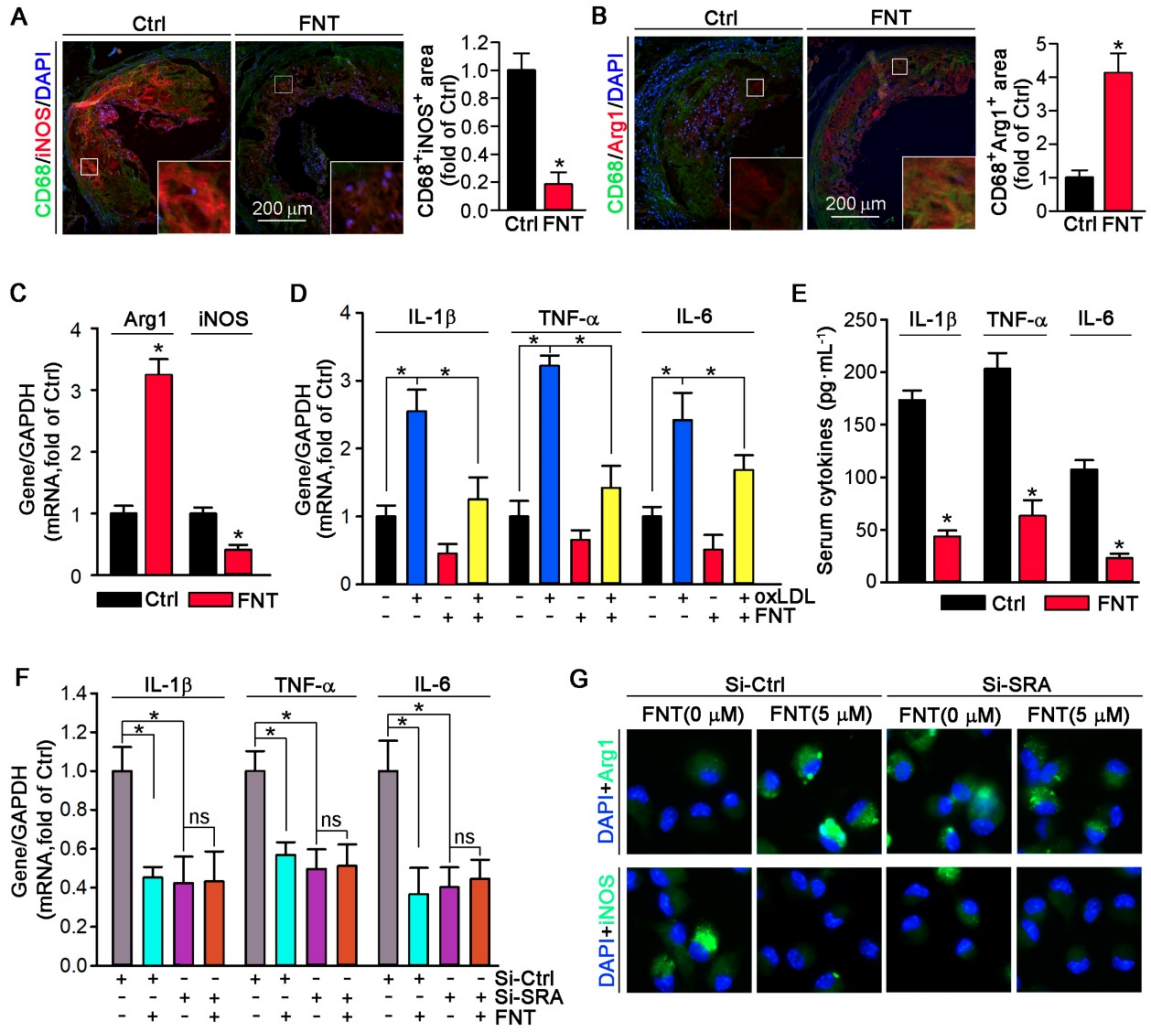
** Formononetin inhibits inflammation and promotes the macrophage M2 transition.** (**A, B**) Aortic root cross sections from mice used in Figure 1 were performed co-immunofluorescent staining with anti-CD68 and iNOS or Arg1 antibodies with quantitative analysis of CD68^+^iNOS^+^ or CD68^+^ Arg1^+^ area, n=5. (**C**) Expression of Arg1 and iNOS mRNA was determined by q-RT-PCR, n=5. (**D**) expression of IL-1β, TNFα and IL-6 mRNA was determined by q-RT-PCR, n=5. (**E**) Serum IL-1β, TNFα and IL-6 was determined by Elisa, n=15. (**F**) After SRA knockdown in PMs, expression of IL-1β, TNFα and IL-6 mRNA was determined by q-RT-PCR, n=5. (**G**) After SRA knockdown in PMs, expression of Arg1 and iNOS was determined by immunofluorescent staining, n=5. Data are presented as mean ± SEM, *P<0.05, significantly different as indicated; ns: not significantly different.

Phenotypic transition of macrophage in atherosclerotic lesion plays a vital role in progress of atherosclerosis. Therefore, we detected the phenotype of macrophage in plaque. Formononetin decreased the iNOS (M1 marker) expression; whereas induced the expression of Arg1 (M2 marker) in macrophage by co-immunofluorescent staining with CD68 (Figure 5A-B). Moreover, the expression of Arg1 in PMs that isolated from formononetin-treated apoE^-/-^ mice was higher than that of control mice; whereas iNOS expression was lower (Figure 5C). Consistent with the effects *in vivo*, formononetin also inhibited oxLDL-induced proinflammatory cytokines, such as TNF-α, IL-1β, and IL-6 at transcriptional level in PMs (Figure 5D). In addition, formononetin reduced LPS-induced ROS production (Figure S4). We also determined that chronic treatment of apoE^-/-^ mice with formononetin substantially reduced serum TNF-α, IL-1β, and IL-6 (Figure 5E). Furthermore, formononetin decreased dendritic cells, NK cells, B cells and T cells contents in the aortic plaque (Figure S5). These results demonstrated that formononetin exerted anti-inflammatory effect *in vivo* and *in vitro*. SRA plays an important role in macrophage phenotypic change 15. Therefore, we further investigated whether the inhibitory effect of formononetin on inflammation is SRA-dependent. After silencing of SRA by siRNA, the effect of formononetin on expression of M1/M2 markers and proinflammatory cytokines were substantially abolished (Figure 5F-G), suggesting that SRA plays a critical role in formononetin-inhibited inflammatory response in atherogenesis.

### Formononetin inhibits SRA expression by increasing KLF4 expression and nuclear translocation

**Figure 6:**
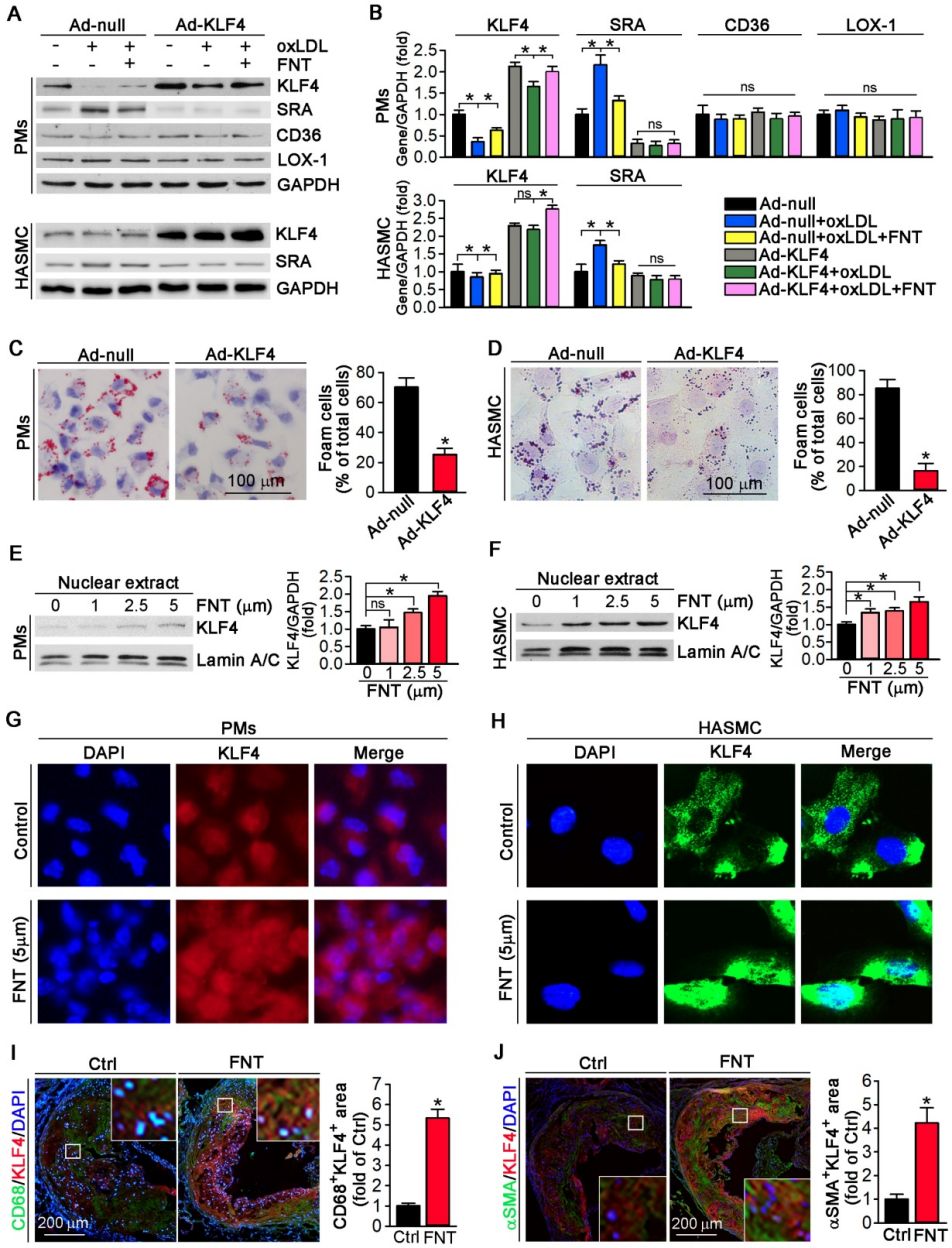
** Formononetin increases KLF4 expression and nuclear translocation in PMs and HASMCs.** (**A, B**) After overexpression of KLF4 by infection with Ad-KLF4, PMs and HASMCs were exposed to oxLDL in presence or absence of formononetin. Expression of KLF4, SRA, CD36 and LOX-1 protein in PMs or KLF4, SRA in HASMCs was determined by Western blot (left) followed by quantification of band density correspondingly (right), n=5. (**C, D**) PMs and HASMCs were stained with Oil Red O after overexpression of KLF4, n=5. (**E, F**) PMs and HASMCs were treated with formononetin at the indicated concentrations overnight. And then expression of KLF4 protein in nuclear extracts was determined by Western blot, respectively, n=5. (**G, H**) Expression of KLF4 protein in PMs and HASMCs was determined by immunofluorescent staining, n=5. (**I, J**) Expression of KLF4 protein in macrophages and VSMCs within plaque was determined by co-immunofluorescent staining with KLF4 and CD68 or αSMA antibodies, n=5. Data are presented as mean ± SEM, *P<0.05; significantly different as indicated; ns: not significantly different.

We utilized KLF4 overexpression by adenovirus (Ad)-KLF4 infection to further delineate the role of KLF4 in regulating SRA expression. PMs and HASMCs were infected with Ad-null or Ad-KLF4 followed by treatment with formononetin. Figure 6A showed that KLF4 expression was markedly increased after Ad-KLF4 infection. Moreover, oxLDL reduced KLF4 expression in both Ad-null and Ad-KLF4 infected cells. However, formononetin antagonized oxLDL-inhibited KLF4 expression (Figure 6A-B). Intriguingly, associated with increment of KLF4 expression in the Ad-KLF4-infected cells, the expression of SRA was correspondingly inhibited (Figure 6A-B), indicating that KLF4 is critical for inhibition of SRA expression by formononetin. In addition, KLF4 overexpression did not affect the expression of CD36 and LOX-1 in PMs (Figure 6A-B), indicating that expression of CD36 and LOX-1 was not KLF4-regulated. We further determined whether KLF4 overexpression can reduce cellular lipid accumulation. Noticeably, overexpression of KLF4 significantly reduced lipid droplets in PMs and HASMCs (Figure 6C-D), which was similarly to that of formononetin (Figure 2C-E) or si-SRA (Figure 3C-D) treated cells, indicating that KLF4 plays a critical role in regulating SRA-mediated cholesterol uptake. As a transcription factor, the nuclear translocation of KLF4 determines its activity of function. Intriguingly, formononetin increased the nuclear expression of KLF4 in PMs and HASMCs (Figure 6E-F), which is consistent with the results obtained by immunofluorescent staining (Figure 6G-H). Moreover, formononetin significantly increased KLF4 expression in macrophages and VSMCs in plaque (Figure 6I-J). Collectively, the results above indicated that KLF4 can negatively regulate SRA expression; and formononetin inhibited the expression of SRA by promoting KLF4 expression and translocation into nucleus.

### KLF4 may act as a transcriptional suppressor on SRA expression

**Figure 7:**
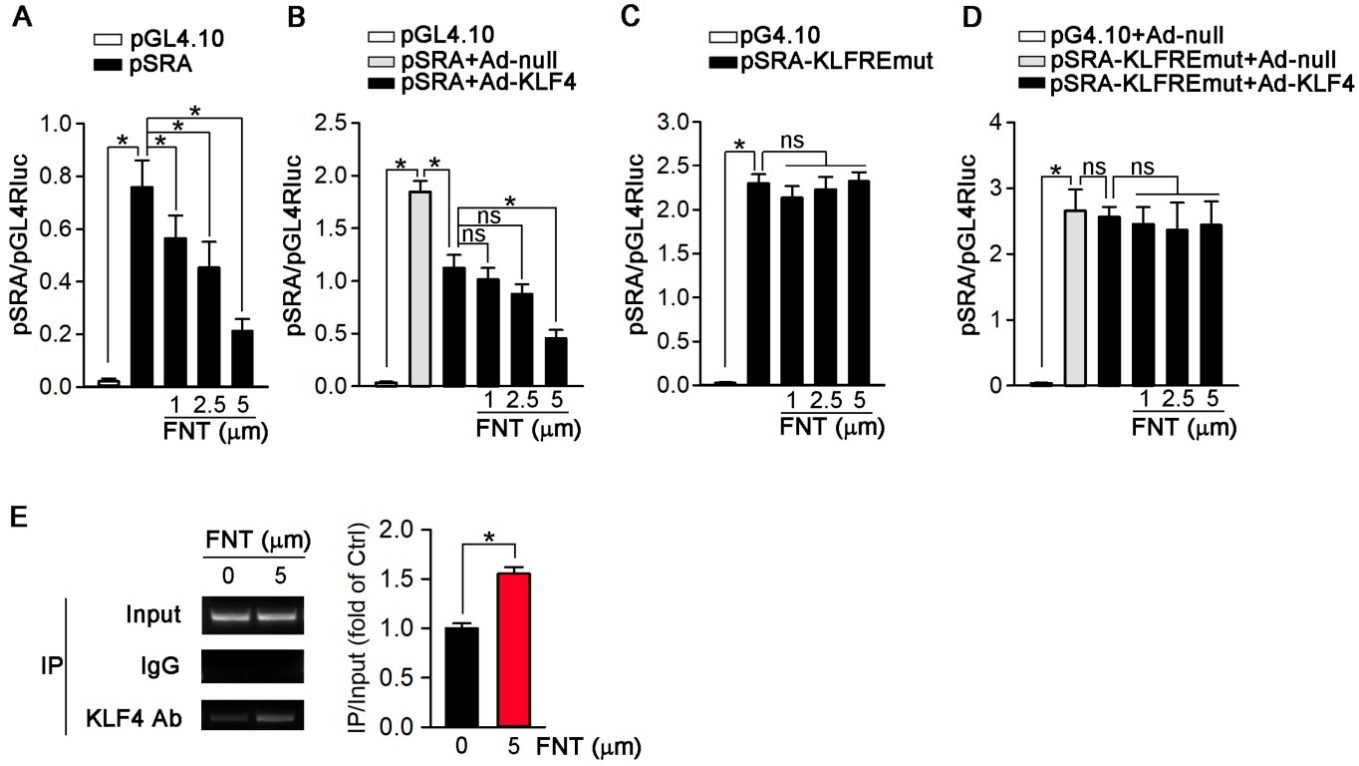
** KLF4 acts as a transcriptional suppressor of SRA.** (**A**) 293T cells were transfected with pGL4.10 vector containing SRA promoter-linked Firefly gene (pSRA) together with pGL4.70 vector containing Renilla gene, and then treated with formononetin for 12 h. The transcriptional activity of SRA was measured by a dual luciferase assay system and normalized to that of Renilla, n=5. (**B**) 293T cells were infected with Ad-KLF4, and then transfected with pSRA together with pGL4.70 vector, followed by treatment with formononetin for 12 h. The transcriptional activity of SRA was measured by a dual luciferase assay system and normalized to that of Renilla, n=5. (**C**) 293T cells were transfected with pGL4.10 vector containing mutant SRA promoter-linked Firefly gene (pSRA-KLFREmut) together with pGL4.70 vector, and then treated with formononetin for 12 h. The transcriptional activity of SRA was measured as described in (B), n=5. (**D**) 293T cells were firstly infected with Ad-KLF4, and then transfected with pSRA-KLFREmut together with pGL4.70 vector, followed by treatment with formononetin for 12 h. The transcriptional activity of SRA was measured as described in (B), n=5. (**E**) After treatment of formononetin, the ability of KLF4 binding to SRA promoter was assayed by the chromosome-immunoprecipitation experiment, n=5. Data are presented as mean ± SEM, *P<0.05; significantly different as indicated; ns: not significantly different.

To further determine if the inhibition of SRA expression occurs at the transcriptional level, we constructed an SRA promoter (pSRA) that included the KLFRE (KLF response element); and determined the promoter activity in response to formononetin treatment. Figure 7A showed that formononetin decreased pSRA activity in a concentration-dependent manner. To further disclose the underlying mechanism, we investigated the effects of KLF4 overexpression on SRA promoter activity. Based on the previously published KLF4 binding sequence (GCGCCCT) 38,39, we found a putative KLFRE in the SRA promoter (GCGCCCT from -111 to -105) through the sequence alignment analysis. The role of KLF4 in formononetin-inhibited SRA transcription was determined by the promotor assay. Intriguingly, as shown in Figure 7B, KLF4 overexpression markedly reduced the activity of SRA promoter, and this reduction was further enhanced by formononetin, indicating that KLF4 can negatively regulate SRA expression. We further constructed mutant SRA promoter with KLFRE mutation (pSRA-KLFREmut) as mutant control. Noticeably, the activity of pSRA-KLFREmut was significantly elevated compared with normal pSRA (~2.3 vs ~0.75) (Figure 7A and 7C), which further indicated that KLF4 may inhibit SRA expression by binding KLFRE. Moreover, the inhibition of SRA promoter activities by KLF4 (Figure 7B) was abolished by KLFRE mutation (Figure 7D); and activities could not be further reduced by formononetin (Figure 7C) or Ad-KLF4 (Figure 7D) after KLFRE mutation, indicating the critical role of the KLFRE in SRA transcription. Furthermore, the binding of KLF4 to KLFRE in SRA promoter was determined by Chip assay (Figure 7E). The results above indicated that KLF4 played a critical role in negatively regulating SRA expression via binding to a KLF-responsive region in the promoter.

## Discussion

Lipid disorder and inflammation are major contributors to foam cell formation during the development of atherosclerosis. Formononetin is a major isoflavone component from *Astragalus membranaceus*. Given that formononetin exhibits multiple inhibitory effects on proatherogenic factors, such as obesity, dyslipidemia, and inflammation in different animal models 35,40,41, we postulate that formononetin may exert antiatherogenic function. However, pharmacological effect and molecular function of formononetin in atherosclerosis remains unknown. According to the references 42,43 and our previous work 24,44, 16 weeks HFD can successfully induce the atherosclerotic model in apoE^-/-^ mice. Therefore, in this study, we used 8 weeks old apoE deficient mice and fed 16 weeks HFD to make atherosclerotic model. Chronic treatment with formononetin resulted in substantial reduction in the atherosclerotic development in the aortic sinus and *en face* in apoE^-/-^mice (Figure 1A-B). Moreover, formononetin effectively improved the lipid profile in the murine model of atherosclerosis (Table 1), which strengthened its antiatherogenic property. The results above indicated its therapeutic potential as a prodrug candidate for atherosclerosis treatment. It's worth noting that mice models accumulate fewer lesions in the coronary arteries, the main sites of atherosclerotic plaque development in humans 45. This may be the limitation in atherosclerosis study using mice models. However, studies with mice models to disclose the molecular mechanisms involved in the development of atherosclerosis provide us invaluable knowledges to explore novel therapeutic strategy.

Increased vulnerability is prone to final rupture of atherosclerotic plaque, which can result in severe cardiovascular event 46. Of note, composition of plaque determines the lesion stability 47. In our study, formononetin significantly decreased the vulnerability index of the atherosclerotic plaque (Figure 1I), suggesting that the potential risk of cardiovascular event may be reduced by formononetin. Moreover, vascular calcification is a major risk factor for vulnerability and even rupture of atherosclerotic plaque. We have reported that inhibition of vascular calcification is a promising approach to inhibit the progress of atherosclerosis 44. Therefore, we evaluated the effect of formononetin on calcium deposition in plaque. In this study, we showed that formononetin significantly reduced the calcium deposition in plaque (Figure 1G-H), which suggest that formononetin may strengthen plaque stability by reducing vascular calcification. However, the underlying mechanism need to be further revealed.

Foam cell formation is a hallmark of atherosclerosis. Several strategies have been exploited to inhibit formation of foam cell to treat atherosclerosis, among which the molecules responsible for cholesterol uptake and efflux are major targets for pharmacological intervention. For instance, ABCA1 and ABCG1 are two molecules in charge of cholesterol efflux from cells to apolipoproteins, triggering which can reduce the intracellular lipid deposition and subsequently retard the foam cell formation 19,48,49. Unfortunately, ABCA1 and ABCG1 are downstream genes of LXR, activation of which can lead severe hepatic steatosis 50. Additionally, VSMC is another major source of foam cell population 51. However, VSMC expresses few ABCA1 and ABCG1 6, which pose an obstacle to the strategy of enhancing cholesterol efflux by ABCA1 and ABCG1. Indeed, despite the level of cholesterol efflux was not changed by formononetin in both PMs and HASMCs, the ability of cholesterol efflux was significantly lower in HASMCs than that of PMs (Figure 3J), which may be attributed to the lower expression of ABCA1 and ABCG1 in HASMCs than in macrophages 6.

In addition, previous studies have reported that the functions of SRA, CD36 and LOX-1 are predominantly involved in molecular roles of cholesterol uptake 52, by which macrophages can ingest oxLDL and then transform into cholesterol-overladen foam cells, subsequently trigger a series of inflammatory responses and thereby promote formation of atherosclerotic plaque 53. Studies have shown that knockout or inhibition of MSR can significantly inhibit the foam cell formation and atherosclerosis 54-56. As is reported, 3-48h oxLDL treatment can induce foam cell formation 24,57. In this study, we incubated the PMs with oxLDL for 24h. Formononetin markedly reduced the lipid burden in PMs (Figure 2C-D) and HASMCs (Figure 2E), which should be attributed to the inhibition of cholesterol uptake (Figure 2G-H) under the condition that cholesterol efflux was not changed (Figure 3J). These results indicated that formononetin can suppress oxLDL-induced foam cell formation from PMs and HASMCs by reducing the cholesterol uptake, without effects on cholesterol removal. Mechanistically, SRA, the classical scavenger in PMs mediating oxLDL uptake, is significantly reduced by formononetin, whereas the expression of ABCA1 and ABCG1 was not affected (Figure 3A-B). Furthermore, the role of SRA in cholesterol uptake in PMs and HASMCs was confirmed by siRNA assay.* In vitro*, knockdown of SRA in PMs and HASMCs resulted in significant reduction in lipid accumulation (Figure 3C-D). Moreover, cholesterol uptake was dramatically decreased after knockdown of SRA (Figure 3E-F), which confirmed the role of SRA in cholesterol uptake. *In vivo*, formononetin markedly inhibited the SRA expression in macrophages and VSMCs of aortic sinus (Figure 3G-H). Moreover, *in vitro*, we found that oxLDL induced HASMCs toward macrophage-like cells, which was characterized with more expression of macrophage marker CD68; and less SMC marker αSMA (Figure 2F and 3I). Intriguingly, formononetin significantly antagonized the effect of oxLDL on HASMCs phenotype switch (Figure 2F and 3I), suggesting that inhibition of VSMC-derived foam cells. The above *in vivo* and associated *in vitro* results suggested that inhibition of SRA by formononetin can reduce foam cell formation and ameliorate atherosclerosis.

Previous studies have reported that SRA can mediate macrophage adhesion 10,58,59, which plays a key role in initiation and development of atherosclerosis. Consistently, in this study, we observed that formononetin significantly inhibited expression of SRA in PMs (Figure 3A-B). More importantly, formononetin inhibited the monocyte adhesion to HUVECs *in vitro* and to aortic endothelium *in ex vivo* (Figure 4A-B). Furthermore, we determined whether inhibition of monocyte adhesion by formononetin was SRA-mediated. Indeed, knockdown of SRA by siRNA reduced the number of monocyte adhesion to HUVECs and aortic endothelium (Figure 4C-D), implying that SRA plays a key role in mediating monocyte adhesion. Noticeable, the reduction of monocyte adhesion by SRA knockdown was not as significant as that of formononetin (Figure 4A-D), indicating that formononetin may affect other molecules responsible for cell adhesion. Such protection beyond inhibition of SRA might be partially attributed to reduction of VCAM-1, ICAM-1 and PECAM-1, three adhesion molecules that can be triggered in dysfunctional endothelium, which can facilitate the adhesion of monocyte to endothelial layer and infiltration 60,61. Accordingly, in this study, formononetin markedly decreased the oxLDL-induced expression of VCAM-1, ICAM-1 and PECAM-1 (Figure 4E), which further enhanced the inhibitory effect on monocyte adhesion by SRA inhibition. These results suggested that inhibition of SRA by formononetin is partially causative for reduced monocyte adhesion and subsequent infiltration in artery wall, which can make contributions to the anti-atherogenic properties of formononetin.

Dysregulation of the M1/M2 phenotypic balance has emerged as a key mechanism involving the pathogenesis of chronic inflammatory diseases, such as atherosclerosis 62,63. Previous study has reported that activation of SRA can promote the M1 polarization, thereby enhancing the development of proinflammatory status 15. Recently, the Canakinumab Anti-inflammatory Thrombosis Outcome Study trial has proven that targeting IL-1β effectively reduces cardiovascular disease risk and mortality in patients with inflammation 64. Intriguingly, in this study, formononetin significantly restrained M1 macrophage polarization while favored the M2 macrophage phenotype *in vivo* and *in vitro* (Figure 5A-C), which may be associated with inhibition of SRA expression in macrophages (Figure 3A); and by which may protect against inflammation and thus limit development of atherosclerosis. Simultaneously, formononetin also markedly reduced the levels of proinflammatory cytokines in PMs and serum (Figure 5D-E). The involvement of SRA in macrophage phenotypic switch was further confirmed by knockdown assay. After knockdown of SRA, PMs are prone to M2 phenotype by determination of M1/M2 and proinflammatory markers (Figure 5F-G). Noteworthy, after SRA knockdown, formononetin cannot further reduce the expression of proinflammatory cytokines, such as IL-1β, TNF-α, and IL-6 (Figure 5F), suggesting that SRA plays a key role in formononetin-inhibited inflammatory response. Therefore, the antiatherogenic effect of formononetin may be partially due to inhibition of inflammatory response during the development of atherosclerosis.

As a zinc finger domain-containing transcription factor, KLF4 involves in atherosclerosis development 27,65. More importantly, it is reported that KLF4 is a critical regulator of macrophage polarization, and expression of KLF4 was robustly induced in M2 macrophages while markedly reduced in M1 macrophages 31. Moreover, in HUVECs, KLF4 negatively regulates expression of VCAM-1 and ICAM-1, which plays a key role in atherosclerotic development 66. Accordingly, in this study, formononetin significantly promoted macrophage toward M2 phenotype by inhibition of SRA expression (Figure 5A-C and 5G) and inhibited the expression of VCAM-1, PECAM-1and ICAM-1 in HUVECs (Figure 4E). These results indicated that the function of formononetin may be partially through KLF4-mediated regulation of SRA expression. Therefore, we further investigated the involving mechanism. In this study, expression of SRA was negatively regulated by KLF4 at translational and transcriptional level (Figure 6A and 7B-D), in which KLF4 functioned as a transcriptional repressor on SRA expression by binding to KLFRE in SRA promoter (Figure 7E). Interestingly, the molecular mechanism of formononetin-inhibited expression of SRA involves increasing KLF4. Notably, formononetin increased the expression and nuclear translocation of KLF4 *in vitro* (Figure 6E-H), and increased expression in macrophages and VSMCs within plaques (Figure 6I-J), by which formononetin significantly reduced SRA expression and lipid accumulation in macrophages and VSMCs, and thus inhibited atherosclerotic development, indicating that KLF4-regulated SRA expression played a key role in foam cell formation and atherogenesis.

In conclusion, our study demonstrated that formononetin can increase KLF4 expression and nuclear translocation, which can, in turn, reduce the expression of SRA in macrophages and HASMCs; by which formononetin inhibits the foam cell formation and inflammatory response during atherogenesis. Formononetin may function as a novel approach to inhibit the development of atherosclerosis.

## Materials and methods

### Cell culture

All cell lines were purchased from ATCC (Manassas, VA). Human aortic smooth muscle cells (HASMCs) were cultured in complete DMEM F12 medium containing 10% FBS, 50 μg mL^-1^ penicillin/streptomycin and 2 mM glutamine. HASMCs (~85% confluence) received treatment in serum-free medium. Human umbilical vein endothelial cells (HUVECs) were cultured in VascuLife basal medium containing VEGF lifeFactors Kit (Lifeline Cell Technology, Frederick, MD). THP-1 cells and peritoneal macrophages were cultured in complete RPMI1640 medium containing 10% FBS, 50 μg mL^-1^ penicillin/streptomycin and 2 mM glutamine.

### *In vivo* studies

The protocol for *in vivo* studies was approved by the Ethics Committee of Tianjin University of Traditional Chinese Medicine and conforms to the Guide for the Care and Use of Laboratory Animals published by the NIH (NIH publication, eighth edition, updated 2011). ApoE^-/-^ mice (males, ~8-week-old, ~22 g bodyweight) with C57BL/6J background were purchased from Beijing Vital River Laboratory Animal Technology Co., Ltd. The animals were housed in SPF units of the Animal Center at Tianjin University of Traditional Chinese Medicine, at 23 ± 1°C, with a relative humidity of 60-70% and a 12 h light/dark cycle. The animals can freely access to water and high-fat diet containing 41% fat plus 0.5% cholesterol (MD12015A, Medicience Ltd., China) during the treatment. The animals were daily checked for food intake, water drink and bodyweight gain during the treatment. ApoE^-/-^ mice were randomly divided into 2 groups (15/group) and fed HFD, HFD containing FNT [10 mg day^-1^ kg^-1^ bodyweight (mpk)] for 16 weeks, respectively. At the end of experiment, all mice were anesthetized and euthanized as we previously reported 24, followed by collection of aortas, peritoneal macrophages (PMs), blood samples and other tissues. Serum was prepared to determine levels of total cholesterol (Total-C), high-density lipoprotein (HDL)-C, low-density lipoprotein (LDL)-C, and triglycerides 24. Serum TNF-α, IL-1β, and IL-6 levels were determined by Elisa assay.

### Atherosclerotic lesion analysis

The aortas were collected and used to prepare aortic root cross sections followed by determination of *en face* and sinus lesions with Oil Red O staining 24. All the images were obtained with a microscope and quantified lesion areas in *en face* aorta and aortic root cross sections, respectively, using a computer-assisted image analysis protocol (Photoshop CS6). The lesion areas were expressed as μm^2^. Necrotic core, fibrous cap, collagen content, calcification, and expression of CD68, αSMA, Arg1, SRA and iNOS protein in lesion areas were determined by Haematoxylin and eosin (H&E), Verhoeff-Van Gieson (VVG), Alizarin Res S and immunofluorescent staining with aortic root cross sections, respectively 24. The calcium content of whole aorta was determined by Calcium LiquiColor kit, as we previously reported 44. The vulnerability index of plaques was calculated as (macrophage staining% + lipid staining%)/(SMCs% + collagen fibre%), according to a previous report 67.

### 3-(4,5-dimethythiazol-2-yl)-2,5-diphenyl tetrazolium bromide (MTT) assay

HASMCs or PMs were seeded into 96-well plates and incubated for 24 h in CO_2_ incubator before cell attachment. Subsequently, cells were switched to medium containing various concentrations of formononetin for another 24 h. After treatment, the cells were incubated with 100 μL of medium with 50 μg MTT (Sigma-Aldrich) for 4 h. Finally, the culture medium was removed, and the formazan precipitates were dissolved in 200 μL of DMSO (Sigma-Aldrich) for determination of absorbance at 595 nm wavelength by a microplate reader (Thermo).

### Determination of foam cell formation *in vitro* and *in vivo*

*In vivo*, PMs were isolated by lavage with PBS from formononetin-treated apoE^-/-^ mice and seeded on cover slips in 24-well plates. The attached cells were fixed by paraformaldehyde and then stained with Oil Red O solution. Cells containing lipid droplets (>10/cell) were considered as foam cells, and >10 fields/sample were counted.

*In vitro*, PMs isolated from untreated apoE^-/-^ mice and HASMCs were seeded on cover slips in 24-well plates. After attachment, PMs and HASMCs in serum-free RPMI1640 and DMEM F12 medium, respectively, were added with oxLDL and incubated for 3 h to induce foam cell formation, followed by formononetin treatment for 16 h. After treatment, cells were fixed by paraformaldehyde and then stained with Oil Red O solution and directly photographed, and lipid burden was quantified by the intensity of extracted ORO dye in DMSO.

### Cholesterol uptake assay

PMs and HASMCs were incubated in medium containing 1,1'-dioctadecyl-3,3,3',3'-tetramethyl indocarbocyanine perchlorate (DiI-oxLDL, Invitrogen) to assay their ability of cholesterol uptake. PMs and HASMCs (1.0×10^6^ cells/well) in 12-well plates were incubated with 10 μg/mL DiI-oxLDL in presence or absence of formononetin for 6 h at 37°C in CO_2_ incubator. Fluorescent images were captured with microscope and then fluorescence intensity was measured by ImageJ.

### Cholesterol efflux assay

PMs and HASMCs incubated with 3-dodecanoyl-NBD cholesterol (1 μg/mL, Cayman Chemical) for 6 h. After incubation, the medium was removed, and cells were washed. And then cells were switched into serum-free medium containing both apo-AI (5 μg/mL) and HDL (20 μg/mL) as cholesterol receptor in the presence or absence of formononetin for 5 h. The fluorescence-tagged cholesterol released from the cells into the medium was measured with an automatic microplate reader (Thermo Scientific, Varioskan Lux, USA). At the same time, cells lysate was prepared for measurement of fluorescence-tagged cholesterol as abovementioned. Cholesterol efflux was expressed as a ratio of fluorescence in the medium to the total amount of fluorescence in cells plus medium.

### Western blot and quantitative real-time PCR

Total cellular and nuclear proteins were extracted from cells 68. Protein expression of ABCA1, ABCG1, KLF4, VCAM-1, ICAM-1, PECAM-1, CD36, LOX-1, αSMA, GAPDH, CD68, SRBI, Lamin A/C, and SRA were determined by Western blot. Total RNA was extracted from cells or aorta followed by determination of mRNA expression by quantitative real-time PCR (q-RT-PCR) with a reverse transcription kit (Vazyme bioteck co., ltd), a SYBR green PCR master mix (DBI, Bioscience), and the primers with sequences listed in Table 2. Expression of IL-1β, TNF-α, IL-6, Arg1 and iNOS mRNA was normalized by GAPDH mRNA in the corresponding samples.

### Determination of the SRA promoter activity

The mouse SRA promoter (from -509 to +43) including KLF4-responsive element (KLFRE, GCGGCCT, located from -111 to -105) was generated by PCR with mouse genomic DNA and the following primers: forward, 5'-CCCTCGAGAGGAGATCATGAGAATTAAT-3' and backward, 5'-CCAAGCTT ATAGTATTTCAGCATCTGGTAC-3'. After the sequence was confirmed, the PCR product was digested with XhoI and HindIII followed by ligation with the pGL4.10 luciferase reporter vector to yield pGL4.10-mSRA (pSRA). The promoter with the KLFRE mutation (*p*SRA-KLFREmut) was constructed with *p*SRA DNA and primers with the corresponding KLFRE mutation: *p*SRA-KLFREmu-F, '5-AGAAAAAAAAAATTTCGGTAACTTTACCCCACTTC-3'; and *p*SRA-KLFREmu-R, 5'-GAAGTGGGGTAAAGTTACCGAAATTTTTTTTTTCT-3'. To analyze SRA promoter activity, 80% confluent 293T cells in 48-well plates were co-transfected with *p*SRA and pGL4.70 plasmid (containing *Renilla* luciferase gene) using Lipofectamine 2000 (Invitrogen). After 24 hours of transfection and treatment, the cells were lysed and collected, which was used to determine the activities of the Firefly and *Renilla* luciferases by the dual-luciferase reporter assay system from Promega (Madison, WI). The Firefly luciferase activity was normalized to that of Renilla luciferase activity.

### Chromatin immunoprecipitation (ChIP) assay

PMs was pretreated with formononetin (5 μM) overnight. ChIP assays were conducted with commercial kits (Abcam, ab500) as previously described 69. After elution and purification, the chromatin DNA was subjected to real time PCR analysis with primers, forward: 5'-GTGAGACAGCGAGACTCCAT-3' and reverse: 5'-CTCTCATCAATGCACAGGTTT-3'.

### Inhibition of SRA by siRNA and overexpression of KLF4 by infection of Ad-KLF4 in PMs and VSMCs

Small interfering RNA (siRNA) against SRA, and scrambled siRNA were purchased from Santa Cruz Biotechnology. PMs and HASMCs were transfected with scrambled or target siRNA using Lipofectamine RNAiMAX (Invitrogen, Grand Island, NE). After 6 h of transfection, the cells were added with equivalent volume of the medium and continued transfection for 24 h. To overexpress KLF4, we infected the PMs and HASMC with adenovirus (Ad)-KLF4. The transfected or infected cells were then switched into complete medium followed by determination of protein expression by Western blot and lipid accumulation by Oil red O staining.

### Monocyte adhesion to endothelial cells and aortic endothelium

HUVECs that plated in 24-well plates and THP-1 cells were treated with formononetin in the presence of oxLDL overnight. Then THP-1 cells were labeled by the carboxyfluorescein succinimidyl ester (CFSE, 5 μmol L^-1^). Aortas isolated from ApoE^-/-^ mice was cleaned of adventitia and prepared as segments, and then treated with oxLDL. The CFSE-labeled THP-1 cells were then added to HUVECs or endothelium of aortic segments and co-incubated for 1 h. After washout, the adherent THP-1 cells to HUVECs or to aortic endothelium were captured with a microscope and counted with ImageJ. The group that treated with only oxLDL was denoted as control, the total adherent cells of which was defined as 1.

### Reagents

Rabbit anti-ICAM-1, VCAM-1, PECAM-1 and GAPDH polyclonal antibodies; rat anti- DC Marker (33D1); and mouse anti-SRA, αSMA, CD36, Arg1, SRBI, LOX-1, NK Cell Marker, CD4, CD22, Mast Cell Chymase and CD68 monoclonal antibody were purchased from Santa Cruz Biotechnology, Inc. (Santa Cruz, CA). Rabbit anti-ABCG1, ABCA1 and Asialo GM1 polyclonal antibodies were purchased from Novus Biologicals (Littleton, CO). Rabbit anti-iNOS polyclonal antibodies were purchased from Proteintech Group, Inc. (Rosemont, IL). Rabbit anti-KLF4 antibody and anti-Lamin A/C monoclonal antibody were purchased from Abcam (Cambridge, MA). Mouse anti-rabbit IgG-R, mouse anti-rabbit IgG-FITC and m-IgGκ BP-FITC antibodies were purchased from Santa Cruz Biotechnology, Inc. (Santa Cruz, CA). Formononetin was purchased from Yuanye (Shanghai, China).

### Statistical analysis

The data and statistical analysis comply with the recommendations on experimental design and analysis in pharmacology 70. All data are expressed as mean ± SEM or mean ± SD. An unpaired Student's t test was used for comparisons between two groups, or One-way ANOVA for comparisons between multiple groups followed by Turkey's method. Significance was accepted when *P* < 0.05.

## Supplementary Material

Supplementary figures and tables.

## Figures and Tables

**Table 1:** Body weight (BM) and serum lipid profile in apoE^-/-^ mice^†^

Treatment	Control	Formononetin
BW(g)	28.24 ± 2.14	25.92 ± 2.52*
Total-C	17.02 ± 3.28	14.45 ± 2.23*
HDL-C	2.18 ± 0.32	2.23 ± 0.28
LDL-C	4.35 ± 1.41	3.26 ± 1.02*
Triglycerides	0.55 ± 0.11	0.29 ± 0.13*

†: Male apoE^-/-^ mice were treated as indicated in Figure 1. Serum samples were prepared to determine the levels of total cholesterol (Total-C), LDL- and HDL-C, and TG (mM). Date are presented as mean ± SD (n=15), *P<0.05 *vs*. control.

**Table 2:** Sequences of primers for q-RT-PCR

GENE	Forward	Backward
*Arg1*	CTTGCGAGACGTAGACCCTG	CTTCCTTCCCAGCAGGTAGC
*IL-1β*	GACCTTCCAGGATGAGGACA	AGCTCATATGGGTCCGACAG
*IL-6*	CTGCAGCCACTGGTTCTGT	CCAGAGCTGTGCAGATGAGT
*TNFα*	TGGCCCAGGCAGTCAGA	GGTTTGCTACAACATGGGCTACA
*iNOS*	GCTTGCCCCTGGAAGTTTCT	CCTCACATACTGTGGACGG
*GAPDH*	ACCCAGAAGACTGTGGATGG	ACACATTGGGGGTAGGAACA

Arg1: Arginase1; IL-1β: interleukin-1β ; TNFα: tumor necrosis factor α; IL-6: interleukin-6; iNOS: inducible nitric oxide synthase; GAPDH: glyceraldehyde-3-phosphate dehydrogenase.

## Abbreviations

IL-1β: interleukin 1β
IL-6: interleukin 6
oxLDL: oxidized LDL
αSMA: α-smooth muscle actin
TNF-α: tumor necrosis factor α
HASMC: human aortic smooth muscle cells
KLF4: krüppel-like factor 4
HUVEC: human umbilical vein endothelial cells
PECAM-1: platelet and endothelial cell adhesion molecule-1
ICAM-1: intercellular adhesion molecule-1
VCAM-1: vascular cell adhesion molecule-1
FNT: formononetin
HFD: high fat diet
PMs: peritoneal macrophages
