# Supplementary material for: Formononetin attenuates atherosclerosis via regulating interaction between KLF4 and SRA in apoE-/- mice
Source: Theranostics. 2020 Jan 1;10(3):1090–106. doi: 10.7150/thno.38115 (PMC6956811; doi:10.7150/thno.38115)
Supplement: Supplementary file 1 — Supplementary figures and tables. [file thnov10p1090s1.pdf]

Supplementary materials

Formononetin attenuates atherosclerosis via regulating interaction between KLF4 and SRA in apoE$^{-/-}$ mice

Running title: Formononetin retards atherosclerotic plaque development

Chuanrui Ma[1,2,3], Ronglin Xia[7], Shu Yang [4], Lipei Liu[5], Jing Zhang[3], Ke Feng[5], Yuna Shang[5], Jingtian Qu[1,2], Lingwei Li[1,2], Ning Chen[8], Shixin Xu[1,2,3], Wenwen Zhang[9], Jingyuan Mao[1,2], Jihong Han[5,6], Yuanli Chen[6], Xiaoxiao Yang[6], Yajun Duan[6], Guanwei Fan [1,2,3]

[1]First Teaching Hospital of Tianjin University of Traditional Chinese Medicine, Tianjin, China; [2]Tianjin Key Laboratory of Translational Research of TCM Prescription and Syndrome, Tianjin, China; [3]State Key Laboratory of Modern Chinese Medicine, Tianjin University of Traditional Chinese Medicine, Tianjin, China; [4]Department of Endocrinology, The Second Clinical Medical College of Jinan University, Shenzhen People's Hospital, Shenzhen, China; [5]College of Life Sciences, Nankai University, Tianjin; China; [6]School of Food and Biological Engineering, Hefei University of Technology, Hefei, China; [7]Tianjin Hospital, Tianjin, China; [8]School of Materials Science and Engineering, Tianjin University, Tianjin, China; [9]Tianjin Central Hospital of Obstetrics and Gynecology, Tianjin, China

Correspondence should be addressed to:

Guanwei Fan, PhD

State Key Laboratory of Modern Chinese Medicine, Tianjin University of Traditional Chinese Medicine, Tianjin, China

No.88, Chang Ling Road, Li Qi Zhuang Jie, Xi Qing District, Tianjin, P.R. China; Tel: 86-22-27987796; Fax: 86-22-27987796; E-mail: guanwei.fan@tjutcm.edu.cn;

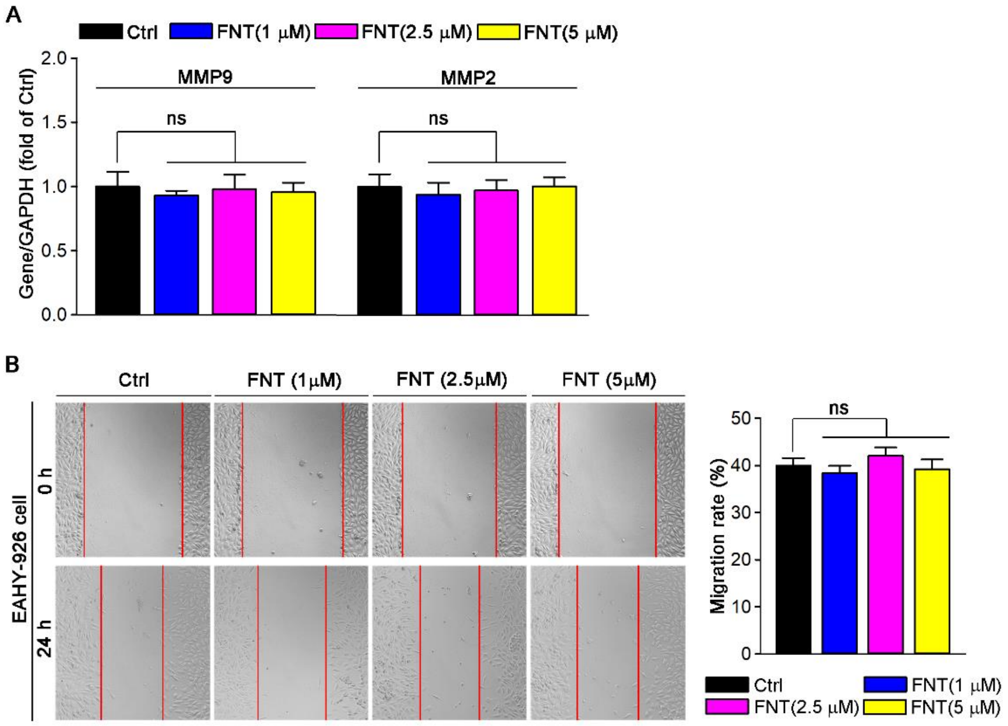

Figure S1. Formononetin does not affect expression of MMP9 /MMP2 and cell migration. (A) EAHY-926 cells were treated with formononetin overnight at the indicated concentrations. After treatment, total RNA was extracted, and expression of MMP9 and MMP2 were determined by q-QT-PCR, n=5. (B) After scratch, EAHY-926 cells were incubated in presence or absence of formononetin for 24 h, and migration rate was evaluated, n=5. Data are presented as mean ± SEM, ns: not significantly different.

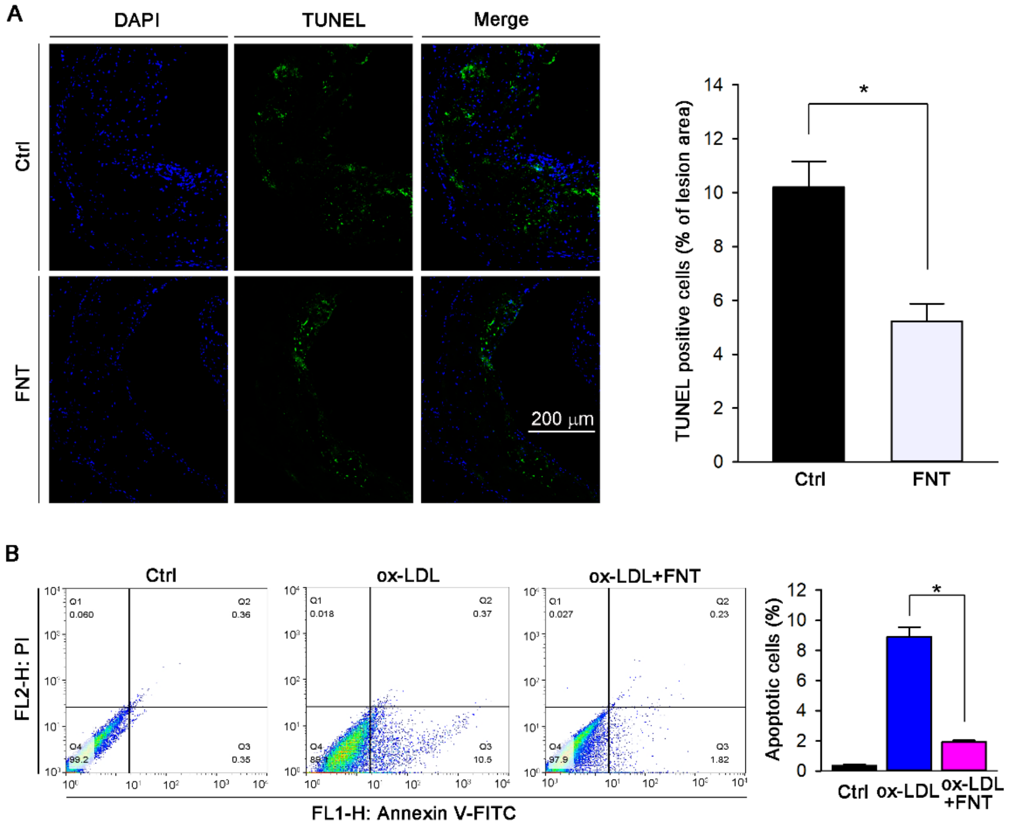

Figure S2. Formononetin decreases the cell apoptosis in lesion plaque and in vitro. (A) Apoptosis in lesion areas of the mice used in Figure 1A by TUNEL staining, n=5. (B) After treatment of ox-LDL (100 μg mL-1) and formononetin (5 μM), macrophages were incubated with FITC Annexin V in buffer containing propidium iodide (PI) and analyzed by flow cytometry. Cells undergoing apoptosis were FITC Annexin V positive and PI negative, n=5. Data are presented as mean ± SEM, *P<0.05, significantly different as indicated.

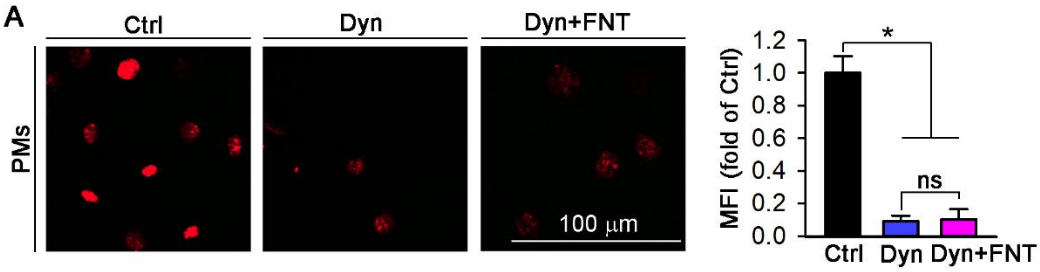

Figure S3. Inhibition of SRA-mediated ox-LDL endocytosis by formononetin may be dynamin-mediated. (A) Peritoneal macrophages (PMs) were incubated with dynamin inhibitor dynasore (Dyn, 1μM) in presence or absence of formononetin (FNT, 5 μM), simultaneously with DiI-oxLDL (10 μg/mL), and then, ability of cellular uptake was assessed, n=5. Data are presented as mean ± SEM, *P<0.05, significantly different as indicated; ns, not significantly.

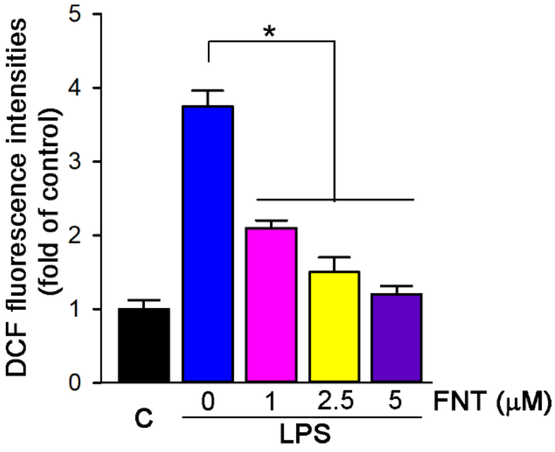

Figure S4. Formononetin reduces cellular ROS levels. Cellular ROS levels in RAW264.7 were determined by DCF staining, n=5. Data are presented as mean ± SEM, *P<0.05, significantly different as indicated.

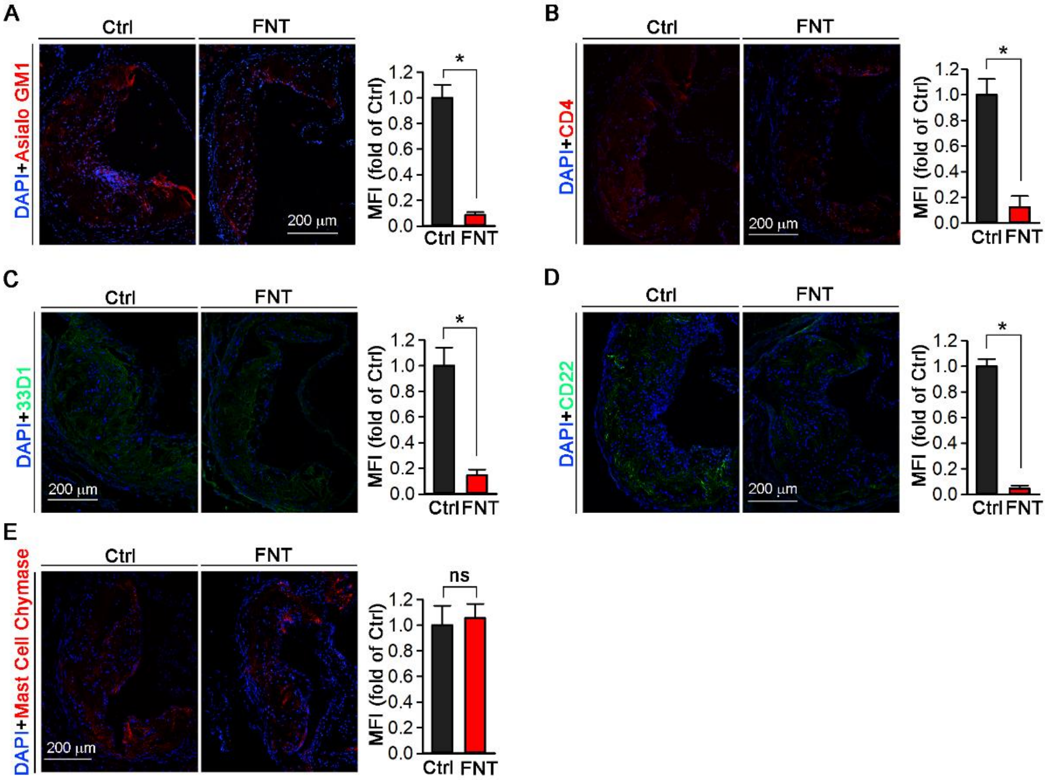

Figure S5. Effect of formononetin on the immune cell profile in lesion plaque. (A-E) Aortic root cross sections from mice used in Figure 1 were performed immunofluorescent staining with anti-Asialo GM1, CD4, CD22, 33D1, Mast Cell Chymase antibodies with quantitative analysis of MFI (mean fluorescent intensity), n=5. Data are presented as mean ± SEM, *P<0.05 versus Ctrl group, ns: not significantly different.
